# Retinal Microvascular Responses to Short-Term Changes in Particulate Air Pollution in Healthy Adults

**DOI:** 10.1289/ehp.1205721

**Published:** 2013-06-18

**Authors:** Tijs Louwies, Luc Int Panis, Michal Kicinski, Patrick De Boever, Tim S. Nawrot

**Affiliations:** 1 Environmental Risk and Health, Flemish Institute for Technological Research (VITO), Mol, Belgium; 2 Centre for Environmental Sciences, and; 3 Transportation Research Institute, Hasselt University, Hasselt, Belgium; 4 Department of Public Health, Leuven University (KU Leuven), Leuven, Belgium

## Abstract

Background: Microcirculation plays an important role in the physiology of cardiovascular health. Air pollution is an independent risk factor for the development and progression of cardiovascular diseases, but the number of studies on the relation between air pollution and the microcirculation is limited.

Objectives: We examined the relationship between short-term changes in air pollution and microvascular changes.

Methods: We measured retinal microvasculature using fundus image analysis in a panel of 84 healthy adults (52% female), 22–63 years of age, during January–May 2012. Blood vessels were measured as central retinal arteriolar/venular equivalent (CRAE/CRVE), with a median of 2 measurements (range, 1–3). We used monitoring data on particulate air pollution (PM_10_) and black carbon (BC). Mixed-effect models were used to estimate associations between CRAE/CRVE and exposure to PM_10_ and BC using various exposure windows.

Results: CRAE and CRVE were associated with PM_10_ and BC concentrations, averaged over the 24 hr before the retinal examinations. Each 10-µg/m^3^ increase in PM_10_ was associated with a 0.93-µm decrease (95% CI: –1.42, –0.45; *p* = 0.0003) in CRAE and a 0.86-µm decrease (95% CI: –1.42, –0.30; *p* = 0.004) in CRVE after adjusting for individual characteristics and time varying conditions such as ambient temperature. Each 1-µg/m^3^ increase in BC was associated with a 1.84-µm decrease (95% CI: –3.18, –0.51; *p* < 0.001) in CRAE.

Conclusions: Our findings suggest that the retinal microvasculature responds to short-term changes in air pollution levels. These results support a mechanistic pathway through which air pollution can act as a trigger of cardiovascular events at least in part through effects on the microvasculature.

Citation: Louwies T, Int Panis L, Kicinski M, De Boever P, Nawrot TS. 2013. Retinal microvascular responses to short-term changes in particulate air pollution in healthy adults. Environ Health Perspect 121:1011–1016; http://dx.doi.org/10.1289/ehp.1205721

## Introduction

Exposure to ambient levels of air pollution increases the incidence of cardiovascular mortality and morbidity (Nawrot et al. 2007; Zanobetti et al. 2003). Research indicates that different fractions of particulate air pollution contribute to the development of cardiovascular disease and provoke cardiovascular events (Brook et al. 2010; Dockery et al. 1993; Nawrot et al. 2011). PM_10_ (particulate matter ≤ 10 µm in diameter) is a complex mixture of compounds including transition metals, sulfate and nitrate salts, and black carbon (Wilson and Suh 1997). Black carbon (BC) is a measure of traffic-related particles that are produced as a combustion by-product.

Although the microcirculation makes up the bulk of the circulatory system, its role in cardiovascular disease remains less clear than the influence of the macrocirculation (Liew et al. 2008). There are two main theories about the significance of microvascular changes in the context of cardiovascular disease. First, microvascular changes could be an early marker for cardiovascular disease, secondary to the disease process (Wong et al. 2004a). Alternatively, microvascular changes could be a primary cause for the development of cardiovascular changes (Levy et al. 2001; Mulvany 1991; Wang et al. 2008). Central retinal arteriolar equivalent (CRAE) is a predictor of future hypertension (Wang et al. 2008). Recent evidence suggests an association between air pollution exposures and markers of microvascular effects (Adar et al. 2010; Barath et al. 2010; Tornqvist et al. 2007).

Changes in the microcirculation can be explored noninvasively by studying retinal blood vessels that are visualized in fundus images (Wong and Mitchell 2007; Wong et al. 2001). The retinal blood vessels have anatomical and physiological features that are comparable with the coronary circulation. Pathologies of the retinal blood vessels parallel changes in the coronary micro- and macrocirculation (Nguyen and Wong 2006; Tedeschi-Reiner et al. 2005; Tso and Jampol 1982). Retinal vessel caliber is an independent predictor for cardiovascular diseases, with arterial narrowing acting as a marker for arteriolar damage and predicting hypertension, and venular widening has been associated with inflammation, endothelial dysfunction, and markers of atherosclerosis (Nguyen and Wong 2006; Wong and Mitchell 2007; Wong et al. 2004a).

Adar et al. (2010) were the first to associate exposure to air pollution with arteriolar narrowing. Among 4,607 participants of the Multi-Ethnic Study of Atherosclerosis (MESA), CRAE narrowed by 0.8 µm (95% CI: –1.1, –0.5) in association with an interquartile increase in long-term exposure [3 µg/m^3^ PM_2.5_ (particulate matter ≤ 2.5 µm in diameter) during the 2 years preceding the clinical exam]. The magnitude of this change corresponded to the change in CRAE associated with a 7-year increase in age in their study population. In a cross-sectional analysis investigating exposure on the previous day, CRAE narrowed by 0.4 µm (95% CI: –0.8, –0.04) in association with a 9-µg/m^3^ increase in PM_2.5_ (Adar et al. 2010).

Our objective was to study the effect of short-term air pollution exposures and microvascular changes in healthy adults (22–63 years of age) using a repeated-measures design.

## Methods

*Study population.* The study was conducted in Belgium during January–May 2012 and included employees of the Flemish Institute for Technological Research (Vlaamse Instelling voor Technologisch Onderzoek; VITO). A total of 183 persons were contacted and 84 (46%) agreed to participate in the study. Participants were 22–63 years of age. All VITO employees undergo an annual clinical examination, and all study participants were free of clinical cardiovascular diseases and diabetes before and during the study period.

Participants were not asked to fast before study visits, and their postprandial status was not recorded. On each study day, participants completed a questionnaire on their current medical history and smoking status; on their use of alcohol, coffee, and specific medications; and on their time spent in traffic during the 24 hr before the clinical visit. Of the 84 persons who participated in our study, 32 (38%) completed one visit, 7 (8%) completed two visits, and 45 (54%) participated in all three clinical visits. The visits were scheduled between 0900 and 1700 hours and took place on the VITO campus. The visits were on average 16 days apart (range, 14–18 days). The clinical visits were scheduled on the same time of day [mean difference, 1.5 hr (range, 0.2–2.2 hr)]. All participants provided written informed consent. The ethics boards of Hasselt University and University Hospital Antwerp approved the study (Hasselt, Antwerpen, Belgium).

*Retinal photography and grading.* The fundus of the right eye of each participant was photographed using a Canon 45° 6.3-megapixel digital nonmydriatic retinal camera (Hospithera, Brussels, Belgium). Participant characteristics were masked for the trained grader before review and analysis of the retinal images. IVAN (Eye Vasculo-matic ala Nicola) retinal image analysis software was used to measure retinal vessel diameters according to previously reported protocols (Hubbard et al. 1999; Knudtson et al. 2003; Wong et al. 2004b). Diameters were summarized as the CRAE and central retinal venular equivalent (CRVE). The equivalents represent a summary of vessel diameters within an area equal to 0.5–1 disc diameters from the optic disc margin.

*Cardiovascular parameters.* Systolic blood pressure (SBP), diastolic blood pressure (DBP), and heart rate (HR) were measured with an automated device (Stabilograph, Stolberg, Germany), according to the guidelines of the European Society of Hypertension (Parati et al. 2008). After the participants rested for 5 min, blood pressure and HR were measured five times consecutively. The average of the last three measurements was calculated and used in data analyses. These cardiovascular parameters were only measured during the second and third clinical examination (*n* = 59).

*Outdoor temperature and barometric pressure.* The 24 hr mean outdoor temperature and barometric pressure measured at the nearby Retie, Belgium, meteorological station (No. 06464; 51°13´50.29˝ N, 5°3´7.64˝ E) were obtained from the Belgian Royal Meteorological Institute (Ukkel, Belgium).

*Air pollution levels: exposure assignment.* Ambient air pollution levels were measured at a nearby official monitoring station in Dessel, Belgium, (No. 42N016; 51°14´2.92˝ N, 5°9´45.58˝ E) and the data were obtained from the Flemish Environmental Agency (Aalst, Belgium). The distance from the monitoring station to the VITO campus is between 5.4 and 9.5 km. The station monitors ambient concentrations of a range of air pollutants, including PM_10_ and BC, every 30 min. PM_10_ was measured with beta-absorption, whereas BC was measured using reflectometry and transmission techniques.

For each participant, average air pollution concentrations were determined for the 2, 4, 6, and 24 hr before the retinal exam (lag 2 hr, 4 hr, 6 hr, and 24 hr, respectively). Air pollution levels were also assigned as a 24 hr average for the previous calendar day (lag 1 day) and 48 hr average for the 2 calendar days preceding the retinal exam (lag 2 day).

*Statistical analysis.* We performed pollutant-specific exposure–response analyses using mixed models that included random effects for each participant across the clinical examinations (SAS, version 9.2; SAS Institute Inc., Cary, NC, USA). This method allows each participant to serve as his or her own control over time and eliminates within-subject confounding by personal characteristics that do not change over time. Associations with exposures over different lag periods (lag 2 hr to lag 2 day) were estimated in separate models. We performed descriptive analyses to identify potential predictors of the markers of the microcirculation that could modify or confound the association between the microcirculation and air pollution exposure. All analyses were adjusted for sex, age, body mass index (BMI), smoking status, alcohol and coffee consumption during the 24 hr prior to the examination, day of the week, time of day, outdoor temperature, and barometric pressure.

In a series of sensitivity analyses, we also adjusted for blood pressure (SBP, DBP) and HR in a subset of 59 participants and adjusted for fellow vessel diameter (i.e., for CRVE in models of CRAE, and vice versa). In addition, we repeated analyses with smokers (*n* = 3) and individuals currently using medication (*n* = 2) excluded. To explore the shape of the dose–response curves, we estimated associations between average PM_10_ concentrations over different lags and the microcirculation markers estimated using unadjusted models with exposures modeled as restricted cubic splines with 5 knots at the 5th, 25th, 50th, 75th, and 95th percentiles (Harrell 2001). Finally, because differences in between- and within-subject air pollution effects could be possible, we fitted separate mixed models that included terms for within- and between-subject exposure effects in addition to the overall model. All tests were two-sided.

## Results

Characteristics of the study population are summarized in Table 1. Of the 84 participants, 52% were women. The population had a mean age of 37 ± 9 years. All participants reported that they were free of diabetes and cardiovascular disease, although one used medication for blood pressure control (an angiotensin receptor blocker) and one used cholesterol-lowering medication (a statin). Three participants were active smokers. All participants had a university or college degree. Short-term air pollution concentrations were highly variable during the study. PM_10_ concentrations (lag 24 hr) ranged from 9.7 to 117.7 µg/m^3^, with interquartile ranges (IQRs) of 9.6, 39.1, and 3.7 µg/m^3^ for the first, second, and third visits, respectively. BC concentrations ranged from 0.37 to 6.99 µg/m^3^, with IQRs of 0.94, 5.64, and 0.29 µg/m^3^ for the first, second, and third visits. During the 5-month study period, the daily outdoor temperature ranged from –6.8 to 20.2°C and the barometric pressure from 993 to 1031 hPa. No within-person correlation was observed for the different exposure periods. Seventy-four participants reported that they spent on average 84 ± 20 min (mean ± SD) driving a car in traffic during the previous 24 hr. Of these 74 participants, 24 reported driving 8 ± 22 min in congested traffic. Twenty-seven participants also reported riding a bicycle in traffic (9 ± 20 min).

**Table 1:** Descriptive characteristics of the study population (*n* = 84).

Characteristic	Mean ± SD or *n* (%)
Age (years)	37 ± 9
Sex
Female (%)	44 (52)
Race/ethnicity
Caucasian (%)	83 (99)
Asian (%)	1 (1)
Smoking status
Current	3 (4)
General health characteristics
BMI (kg/m²)	23 ± 3
SBP^*a*^ (mm Hg)	126 ± 11
DBP^*a*^ (mm Hg)	75 ± 8
HR^*a*^ (bpm)	72 ± 13
Participation in traffic on day of examination
Persons using a car	74 (88)
Persons using a car in congested traffic	24 (30)
Persons riding a bike or walking in traffic	27 (32)
^***a***^Data were available for 59 participants and reported blood pressure values are based on the average of three consecutive readings at two examination moments.	

*Predictors and correlates of CRAE and CRVE.* CRAE and CRVE averaged 136 ± 14 µm and 189 ± 18 µm, respectively. The CRAE/CRVE ratio was 0.722 ± 0.067. CRAE did not differ significantly between men and women (*p* = 0.95), but decreased by 0.59 µm (95% CI: –0.94, –0.23; *p* = 0.0015) in association with a 1-year increase in age. BMI (*p* = 0.97), alcohol use (*p* = 0.58), coffee consumption (*p* = 0.28), outdoor temperature (*p* = 0.58), and barometric pressure (*p* = 0.97) were not significant predictors of CRAE, nor was time of day (*p* = 0.34). A 10-min increase in the amount of time spent in driving a car was associated with a 0.14-µm decrease (95% CI: –0.35, 0.07; *p* = 0.18) in CRAE. Finally, a 1-µm increase in CRVE was associated with a 0.40-µm increase in CRAE (95% CI: 0.30, 0.51; *p* < 0.0001). Outdoor temperature was the only statistically significant predictor of CRVE (0.98-µm decrease with a 1°C increase in outdoor temperature, 95% CI: –1.33, –0.45; *p* = 0.0001).

**Figure 1:**
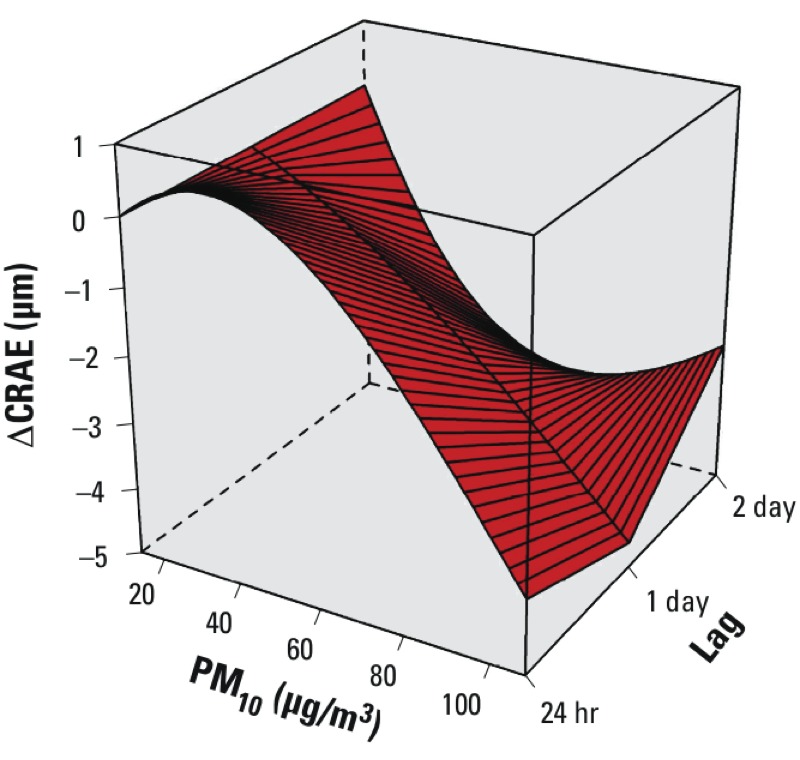
Microvascular responses associated with short-term changes in air pollution shown by unadjusted analysis of change in CRAE after PM_10_ exposure. The effect was estimated using restricted cubic splines with 5 knots located at the 5th, 25th, 50th, 75th, and 95th percentiles for exposures on the day of the examination using average exposure 24 hr before the clinical measurements (lag 24 hr), and on the 24 hr average of the day before (lag 1 day) and 48 hr average of the two preceding days (lag 2 day).

*Microcirculatory markers in association with changes in short-term air pollution.* Unadjusted models of associations between CRAE and PM_10_ modeled using restricted cubic splines did not indicate a threshold effect (Figure 1). An increase in PM_10_ within the low concentration ranges (< 30 µg/m^3^) was associated with a decrease in CRAE for lag 1 day and lag 2 day. Studying the shape of the association showed no threshold effect at higher concentrations and a linear shape (at lag 24 hr at ≥ 30 µg/m^3^) over the full exposure range (Figure 1).

After adjustment for sex, age, BMI, smoking, alcohol and coffee consumption 24 hr prior to the examination, time of the day, day of the week, 24 hr mean outdoor temperature, and barometric pressure, CRAE was associated inversely with the PM_10_ and BC concentration in the hours and days before the clinical examination (Table 2). Each 10-µg/m^3^ increase in average PM_10_ during the previous 24 hr was associated with a 0.93-µm decrease (95% CI: –1.42, –0.45; *p* = 0.0003) in CRAE (Table 2, model 1). Significant negative associations were also estimated between CRAE and average PM_10_ over shorter exposure windows, and for PM_10_ averaged over the previous 2 days. A 1-µg/m^3^ increase in BC during the previous 24 hr also was negatively associated with CRAE (–1.84 µm; 95% CI: –3.18, –0.51; *p* = 0.008), but associations with shorter and longer exposure periods were not significant (Table 2, model 1). All associations with CRAE moved toward the null when adjusted for CRVE in addition to the other covariates (Table 2, model 2); however, statistically significant negative associations persisted for 24 hr average exposures to both PM_10_ and BC.

**Table 2:** Estimated change in mean CRAE (95% CI) in association with a 10-µg/m^3^ increase in PM_10_ or a 1-µg/m^3^ increase in BC.

Exposure time (lags)	Model 1^*a*^	Model 2^*b*^
PM_10_ (for each 10-μg/m³ increase)
2 hr	–0.62 (–1.13, –0.11)*	–0.38 (–0.85, 0.08)
4 hr	–0.67 (–1.22, –0.13)*	–0.41 (–0.90, 0.09)
6 hr	–0.75 (–1.31, –0.18)*	–0.43 (–0.94, 0.09)
24 hr	–0.93 (–1.42, –0.45)^†^	–0.57 (–1.01, –0.12)*
2 day	–0.60 (–1.18, –0.02)*	–0.15 (–0.70, 0.40)
BC (for each 1-μg/m³ increase)
2 hr	0.24 (–0.57, 1.05)	–0.03 (–0.75, 0.69)
4 hr	0.38 (–0.49, 1.26)	0.03 (–0.75, 0.82)
6 hr	0.52 (–0.47, 1.51)	0.10 (–0.79, 0.99)
24 hr	–1.84 (–3.18, –0.51)**	–1.54 (–2.69, –0.39)*
2 day	–0.21 (–1.13, 0.71)	–0.16 (–1.00, 0.68)
Both models include 84 persons: 25 had one measurement, 14 had 2 measurements, and 45 had 3 measurements. ^***a***^Adjusted for sex, age, BMI, smoking habits, alcohol and coffee consumption 24 hr prior to examination, time of the day and day of the week, outdoor temperature, and barometric pressure. ^***b***^Includes model 1 covariates plus CRVE. *p < 0.05. **p < 0.01. ^†^*p *< 0.001.		

CRVE was negatively associated with a 10-µg/m^3^ increase in PM_10_ during the previous 24 hr (–0.86 µm; 95% CI: –1.42, –0.30; *p* = 0.004) and with PM_10_ exposure during other lag periods (Table 3, model 1). A 1-µg/m^3^ increase in BC during the previous 24 hr was also negatively associated with CRVE, although the association was not significant (–1.18 µm; 95% CI: –3.11, 0.75; *p* = 0.23). Most associations moved closer to the null after adjustment for CRAE.

**Table 3:** Estimated change in CRVE in association with PM_10_ and BC. Estimates express the change (95% CI) in the retinal venular blood vessels associated with a 10-µg/m^3^ increase in PM_10_ or a 1-µg/m^3^ increase in BC.

Exposure time (lags)	Model 1^*a*^	Model 2^*b*^
PM_10_ (for each 10-μg/m³ increase)
2 hr	–0.62 (–1.28, 0.04)	–0.39 (–1.00, 0.22)
4 hr	–0.77 (–1.48, –0.05)*	–0.49 (–1.15, 0.17)
6 hr	–0.93 (–1.67, –0.17)*	–0.60 (–1.28, 0.09)
24 hr	–0.86 (–1.42, –0.30)**	–0.60 (–1.26, 0.07)
2 day	–1.03 (–1.88, –0.18)*	–0.84 (–1.61, –0.08)*
BC (for each 1-μg/m³ increase)
2 hr	0.46 (–0.65, 1.57)	0.29 (–0.71, 1.31)
4 hr	0.52 (–0.68, 1.73)	0.30 (–0.80, 1.40)
6 hr	0.47 (–0.87, 1.80)	0.22 (–1.01, 1.44)
24 hr	–1.18 (–3.11, 0.75)	–0.04 (–1.77, 1.70)
Both models include 84 persons: 25 had one measurement, 14 had 2 measurements, and 45 had 3 measurements. ^***a***^Adjusted for sex, age, BMI, smoking habits, alcohol and coffee consumption 24 hr prior to examination, time of the day and day of the week, outdoor temperature, and barometric pressure. ^***b***^Includes model 1 covariates plus CRAE. *p < 0.05. **p < 0.01.		

*Sensitivity analyses.* We did not find statistically significant associations between PM_10_ or BC and blood pressure components (SBP, DBP, or pulse pressure) in the subset of 59 participants with blood pressure data (see Supplemental Material, Table S1). When we adjusted for SBP, DBP, and HR in addition to model 1 covariates and CRAE or CRVE, only the association between lag 24 hr PM_10_ and CRAE was significant (–0.50 µm; 95% CI: –0.92, –0.08; *p* = 0.005) although a 1-µg/m^3^ increase in lag 24 hr BC was also negatively associated with CRAE (–1.08 µm; 95% CI: –2.21, 0.04; *p* = 0.059) (see Supplemental Material, Table S2). No significant associations between CRVE and air pollution indicators were estimated based on this model.

Associations between CRAE and lag 24 hr average PM_10_ and BC persisted when we also adjusted for time spent in traffic, and when we excluded the three smokers and two participants on antihypertensive and/or cholesterol medication (data not shown). The negative associations with lag 24 hr PM_10_ and BC were also confirmed when we excluded the 32 participants who had only one CRAE measurement (*n* = 52) [estimated mean decreases of 0.76 µm (95% CI: –1.32, –0.20; *p* = 0.01) and 1.37 µm (95% CI: –2.90, 0.15; *p* = 0.07) for a 10-µg/m^3^ increase in lag 24 hr PM_10_ and a 1-µg/m^3^ increase in BC, respectively]. Associations were of approximately the same magnitude (although no longer significant) when data from the second set of study visits, which took place during a time of relatively high PM_10_ and BC concentrations, were excluded (data not shown).

Finally, we ran models to differentiate between the within- and between-subject effects. Our overall estimates for PM_10_ were driven by the within-subject effects. Within-subject effect estimates indicated that each 10-µg/m^3^ increase in lag 24 hr PM_10_ was associated with a 0.66-µm decrease in mean CRAE (95% CI: –1.02, –0.30; *p* = 0.0005) and each 1-µg/m^3^ increase in lag 24 hr BC was associated with a 1.08-µm decrease in CRAE (95% CI: –2.02, –0.13; *p* = 0.03) (see Supplemental Material, Table S3). Corresponding estimates for between-subject effects were –1.34 (95% CI: –2.82, 0.13; *p* = 0.07) and –3.68 (95% CI: –6.33, –1.02; *p* = 0.007), respectively.

## Discussion

We found a decrease in CRAE in association with exposure to PM_10_ and BC in a panel of healthy adults. These results remained significant after adjustment for sex, age, BMI, SBP, and DBP or any of the other covariates studied. Arteriolar narrowing is an independent predictor of risk of myocardial infarction, hypertension, and cardiovascular mortality (Cheung et al. 2007a, 2007b; Wong et al. 2002, 2006b).

Other authors have reported an association between blood pressure and acute changes in air pollution (Hoffmann et al. 2012; Jacobs et al. 2012; Wu et al. 2013). Despite the decrease in retinal arteriolar vessel diameter, we did not observe statistically significant associations between PM_10_ or BC and blood pressure in the subset of participants with blood pressure data. We propose three explanations for this lack of association in our study.

Blood pressure is a highly variable phenotype that is regulated by several control mechanisms counteracting changes in vessel diameter (Brook et al. 2002). The present study might not have sufficient power to detect such an effect.

The small vasoconstriction in the retinal blood vessels might not change overall peripheral resistance, thus blood pressure levels remain normal.

Microvascular changes can be a cause or a consequence of elevated blood pressure. In our healthy population, air pollution exposure was associated with microvascular changes after adjustment for blood pressure. The microvasculature might rather be a target for primary changes that could eventually result in elevated blood pressure rather than vice versa. This is in agreement with the hypothesis that microvascular changes can be a primary cause for the development of cardiovascular changes (Levy et al. 2001; Mulvany 1991; Wang et al. 2008). In another study, inhalation of air pollution was associated with acute vasoconstriction of the forearm conduit artery without changes in systemic blood pressure (Brook et al. 2002).

Both fellow vessel diameter and blood pressure components are known to influence the microvascular changes in the retina (Cheung et al. 2007a, 2007b; Wong et al. 2002, 2006b). The effect estimates were attenuated by adjusting for fellow vessel diameter (i.e., including CRVE in models of associations between the exposures and CRAE, and vice versa) (Table 2 and Table 3, model 2), and much less by blood pressure (see Supplemental Material, Table S2). Additional research is needed to clarify the relation between the pollutants, blood pressure, and CRAE or CRVE.

It is likely that both vessel diameters are affected by an identical mechanism and respond in the same way (Liew et al. 2007; Miller et al. 2012). Because of their proximity, these blood vessels could interact by exchanging biologically active agents (Kavdia and Popel 2006). A model that accounts for fellow vessel diameter represents the independent effects of air pollution on both vessels (CRAE/CRVE) but, because of their correlation, overadjustment cannot be excluded.

Exposure to air pollution has been associated with markers of pulmonary inflammation, which can cause a low-grade, systemic inflammation (Chuang et al. 2007; Hoffmann et al. 2009). Inflammation has been linked with endothelial dysfunction (Stenvinkel 2001). The effects of the systemic inflammation reaction may take some time to affect the retinal blood vessels. We hypothesize that inflammatory responses alter the activity of the endothelium and initiate endothelial dysfunction, which may result in the narrowing of the retinal arterioles, even several hours after exposure. Given the high variation in ambient air pollution levels, with intermittent peak episodes, the microvasculature is constantly adapting to a changing environment. Our findings suggest that this might occur very quickly, even ≤ 24 hr. In our first model, exposure to PM_10_ during all the hourly exposure windows was inversely associated with CRAE.

To our knowledge, only Adar et al. (2010) have previously published a study of short-term effects of air pollution on the human retinal microvasculature. The microvascular changes we report here complement those found by Adar et al. (2010), who reported changes in the retinal microcirculation associated with long-term exposure (averaged over the previous 2 years) and short-term exposure (averaged over the previous day) in a cross-sectional analysis using the MESA cohort. Assuming that 9 µg/m^3^ PM_2.5_ corresponds to 12.9 µg/m^3^ PM_10_ (Nawrot et al. 2011), the short-term cross-sectional association reported by Adar et al. (2010) (–0.4 µm; 95% CI: –0.8, –0.04) per 9-µg/m^3^ increase in average PM_2.5_ on the previous day is smaller than our estimate based on repeated measurements (–1.20 µm; 95% CI: –1.61, –0.61). The effect size reported in our study may be larger than the one reported for the MESA cohort because our study population was exposed to greater variation in PM_10_ and BC concentrations. Furthermore, our study population consisted of young, healthy persons, all with the same socioeconomic status, in contrast with the much older and more diverse MESA cohort. In theory, arteriolar narrowing in response to air pollution in healthy persons might be more pronounced than in susceptible persons. A healthy microvasculature may respond better to changing conditions. This healthy response could result in bigger microvascular changes, whereas the response in susceptible persons or persons at risk might be compromised due to their already affected microvasculature.

Our results are consistent with previously reported health effects of air pollution. Toxicological studies have revealed that short-term exposure to peak levels of air pollutants is associated with microvascular responses. Animal studies conducted by Nurkiewicz et al. (2004, 2006, 2011) demonstrated that exposure to (ultrafine) particulate matter induced oxidative stress that led to eNOS (endothelial nitric oxide synthase) uncoupling and reduced bioavailability of the vasodilator NO (nitric oxide). In addition, controlled-exposure studies of humans have reported evidence of impaired macrovascular endothelial function in response to diesel exhaust (Barath et al. 2010; Tornqvist et al. 2007).

Existing evidence suggests that air pollution can trigger an acute autonomic imbalance, favoring sympathetic nerve activity to the smooth muscles surrounding blood vessels (Pieters et al. 2012). Increased sympathetic activity causes smooth muscle contraction and thus vasoconstriction. Retinal blood vessels lack functional sympathetic innervations (Riva et al. 1986); therefore, autonomic imbalance is not likely to be the primal cause of retinal arteriolar vasoconstriction. This might also explain why microvascular changes were more pronounced for the 24 hr exposure window than for the shorter lags.

Previously reported experiments on forearm conduit arteries allow the assessment of endothelial function, but the retinal blood vessels share more similarities in development and anatomy with the microvasculature of the heart, lungs, and brain (Wong et al. 2006a). Therefore, changes in retinal blood vessels may be related to changes in the systemic microcirculation.

Our findings may not be generalizable to the adult population as a whole. Subsequent research should therefore aim at confirming the observations in larger and more diverse populations. In addition, it would be informative to study populations that may be more susceptible to microvascular effects of air pollutants due to underlying pathologies that promote chronic inflammation. Persons with diabetes, for example, have been shown to be vulnerable to the effects of air pollution (Jacobs et al. 2010; von Klot et al. 2005).

We cannot exclude some exposure misclassification. Measurements from a monitoring station close to the study site were used to estimate exposures. However, participants may have been exposed to different BC concentrations at their places of residence or while commuting (Dons et al. 2012, 2013). The amount of time spent driving in traffic, as determined from the questionnaire, was negatively associated with arteriolar diameter, although the association was not statistically significant. Ideally, personal measurements of BC should be used in future studies.

## Conclusions

The key finding of our repeated measurements study in a panel of healthy adults is that an acute narrowing of retinal arterial vessels, a marker for arteriolar damage, was associated with particulate matter air pollution. Based on our analysis, the estimated effect on CRAE associated with a 10-µg/m^3^ increase in average PM_10_ during the 24 hr before the retinal examination was equivalent to the change in CRAE associated with a 1.5-year increase in age. This microvascular response to air pollution might contribute to the development or progression of cardiovascular diseases and complications, as seen in epidemiological studies. Our findings add new evidence to the cardiovascular health effects of short-term exposure to air pollution in healthy persons and suggest a mechanistic pathway through which air pollution can act as a trigger of cardiovascular events at least in part through effects on the microvasculature.

## Supplemental Material

(205 KB) PDF
