# Supplementary material for: Retinal Microvascular Responses to Short-Term Changes in Particulate Air Pollution in Healthy Adults
Source: Environ Health Perspect. 2013 Jun 18;121(9):1011–6. doi: 10.1289/ehp.1205721 (PMC3764070; doi:10.1289/ehp.1205721)
Supplement: (205 KB) PDF [file ehp.1205721.s001.pdf]

Environmental Health Perspectives doi:10.1289/ehp.1205721

Supplemental Material

Retinal Microvascular Responses to Short-Term Changes in Particulate Air Pollution in Healthy Adults

Tijs Louwies, Luc Int Panis, Michal Kicinski, Patrick De Boever, and Tim S. Nawrot

Table of Contents

Page 2: Supplemental Material, Table S1 - Change in blood pressure components (SBP, DBP and PP) in association with particulate air pollution (PM_{10}) and black carbon (BC).

Page 3: Supplemental Material, Table S2 - Change in Central Retinal Arteriolar or Venular Equivalent (CRAE or CRVE) in association with particulate air pollution (PM_{10}) and black carbon (BC).

Page 4: Supplemental Material, Table S3 - Change in Central Retinal Arteriolar Equivalent (CRAE) in association with particulate air pollution (PM_{10}) and black carbon (BC).

Supplemental Material, Table S1. Change in blood pressure components (SBP, DBP and PP) in association with particulate air pollution (PM_{10}) and black carbon (BC). Estimates express the change (95% Confidence Intervals) in the blood pressure component associated with a 10-µg/m³ increase in PM_{10} or a 1-µg/m³ increase in BC.

Exposure Time (lags)	SBP, mmHg	DBP, mmHg	PP, mmHg
PM_{10} (for each 10-µg/m³ increase)			
2 hours	-0.13 (-0.42; 0.16)	-0.05 (-0.18; 0.07)	-0.07 (-0.26; 0.13)
4 hours	-0.17 (-0.48; 0.14)	-0.05 (-0.19; 0.08)	-0.09 (-0.30; 0.11)
6 hours	-0.21 (-0.54; 0.12)	-0.07 (-0.21; 0.07)	-0.13 (-0.35; 0.09)
24 hours	-0.12 (-0.40; 0.17)	-0.001 (-0.12; 0.17)	-0.08 (-0.27; 0.11)
2 days	-0.22 (-0.47; 0.04)	-0.04 (-0.22; 0.12)	-0.16 (-0.34; 0.02)
BC (for each 1-µg/m³ increase)			
2 hours	-0.02 (-0.46; 0.42)	-0.05 (-0.23; 0.14)	0.03 (-0.26; 0.32)
4 hours	-0.05 (-0.53; 0.44)	-0.06 (-0.26; 0.15)	0.02 (-0.30; 0.34)
6 hours	-0.07 (-0.62; 0.48)	-0.06 (-0.30; 0.18)	0.02 (-0.35; 0.39)
24 hours	-0.04 (-0.66; 0.74)	0.05 (-0.12; 0.22)	0.01 (-0.44; 0.46)
2 days	-0.13 (-0.70; 0.44)	0.086 (-0.15; 0.33)	-0.11 (-0.50; 0.29)

Estimates were adjusted for gender, age, BMI, heart rate, smoking habits, alcohol and coffee consumption 24 hours prior to examination, time of the day and day of the week, outdoor temperature and barometric pressure. All models include 59 persons.

Supplemental Material, Table S2 Model 3. Change in Central Retinal Arteriolar or Venular Equivalent (CRAE or CRVE) in association with particulate air pollution (PM_{10}) and black carbon (BC). Estimates express the change (95% Confidence Intervals) in the retinal arteriolar or venular blood vessel diameters associated with a 10-µg/m³ increase in PM_{10} or a 1-µg/m³ increase in BC.

Exposure Time (lags)	**CRAE, µm**	**CRVE, µm**
PM_{10} (for each 10-µg/m³ increase)		
2 hours	-0.25 (-0.68; 0.17)	-0.19 (-0.80; 0.42)
4 hours	-0.30 (-0.75; 0.16)	-0.26 (-0.92; 0.40)
6 hours	-0.32 (-0.79; 0.15)	-0.34 (-1.03; 0.35)
24 hours	-0.50 (-0.92; -0.08)*	-0.39 (-1.06; 0.28)
2 days	-0.40 (-0.95; 0.15)	-0.79 (-1.61; 0.02)
BC (for each 1-µg/m³ increase)		
2 hours	0.15 (-0.54; 0.84)	0.29 (-0.73; 1.31)
4 hours	0.20 (-0.55; 0.96)	0.33 (-0.79; 1.44)
6 hours	0.29 (-0.58; 1.16)	0.34 (-0.92; 1.60)
24 hours	-1.08 (-2.21; 0.04)	0.26 (-1.51; 2.03)
2 days	-0.33 (-1.24; 0.57)	-0.19 (-0.80; 0.42)

In model 3, estimates were adjusted for: gender, age, BMI, smoking habits, alcohol and coffee consumption 24 hours prior to examination, time of the day and day of the week, outdoor temperature and barometric pressure, systolic and diastolic blood pressure, heart rate and fellow vessel diameter. Statistical differences are expressed as: * <0.05, ** <0.01, *** <0.001. Model 3 includes 59 persons.

Supplemental Material, Table S3. Change in Central Retinal Arteriolar Equivalent (CRAE) in association with particulate air pollution (PM_{10}) and black carbon (BC). Estimates express the between- and the within-subject effects (95% Confidence Intervals) on the retinal arteriolar vessel diameters associated with a 10-µg/m³ increase in PM_{10} or a 1-µg/m³ increase in BC.

Exposure Time (lags)	**Between-subject effect**	**Within-subject effect**
PM_{10} (for each 10-µg/m³ increase)		
2 hours	-0.82 (-2.08; 0.40)	-0.33 (-0.64; -0.03)*
4 hours	-0.76 (-2.07; 0.55)	-0.35 (-0.66; -0.03)*
6 hours	-0.81 (-2.11; 0.48)	-0.36 (-0.68; -0.04)*
24 hours	-1.34 (-2.82; 0.13)	-0.66 (-1.02; -0.30)***
2 days	-0.37 (-1.61; 0.87)	-0.19 (-0.65; 0.26)
BC (for each 1-µg/m³ increase)		
2 hours	-0.72 (-2.45; 1.02)	-0.10 (-0.71; 0.50)
4 hours	-0.56 (-2.36; 1.24)	-0.07 (-0.70; 0.56)
6 hours	-0.64 (-2.47; 1.18)	-0.06 (-0.74; 0.61)
24 hours	-3.68 (-6.33; -1.02)**	-1.08 (-2.02; -0.13)*
2 days	-0.21 (-2.12; 1.70)	0.16 (-0.37; 0.69)

Estimates were adjusted for: gender, age, BMI, smoking habits, alcohol and coffee consumption 24 hours prior to examination, time of the day and day of the week, outdoor temperature and barometric pressure. Statistical differences are expressed as: * <0.05, ** <0.01, *** <0.001.
